# Supplementary material for: The G-protein coupled receptor OXER1 is a tissue redox sensor essential for intestinal epithelial barrier integrity
Source: bioRxiv. 2025 Feb 8:2025.02.05.636712. Preprint. [Version 1] doi: 10.1101/2025.02.05.636712 (PMC11839128; doi:10.1101/2025.02.05.636712)
Supplement: Supplement 1 [file NIHPP2025.02.05.636712v1-supplement-1.pdf]

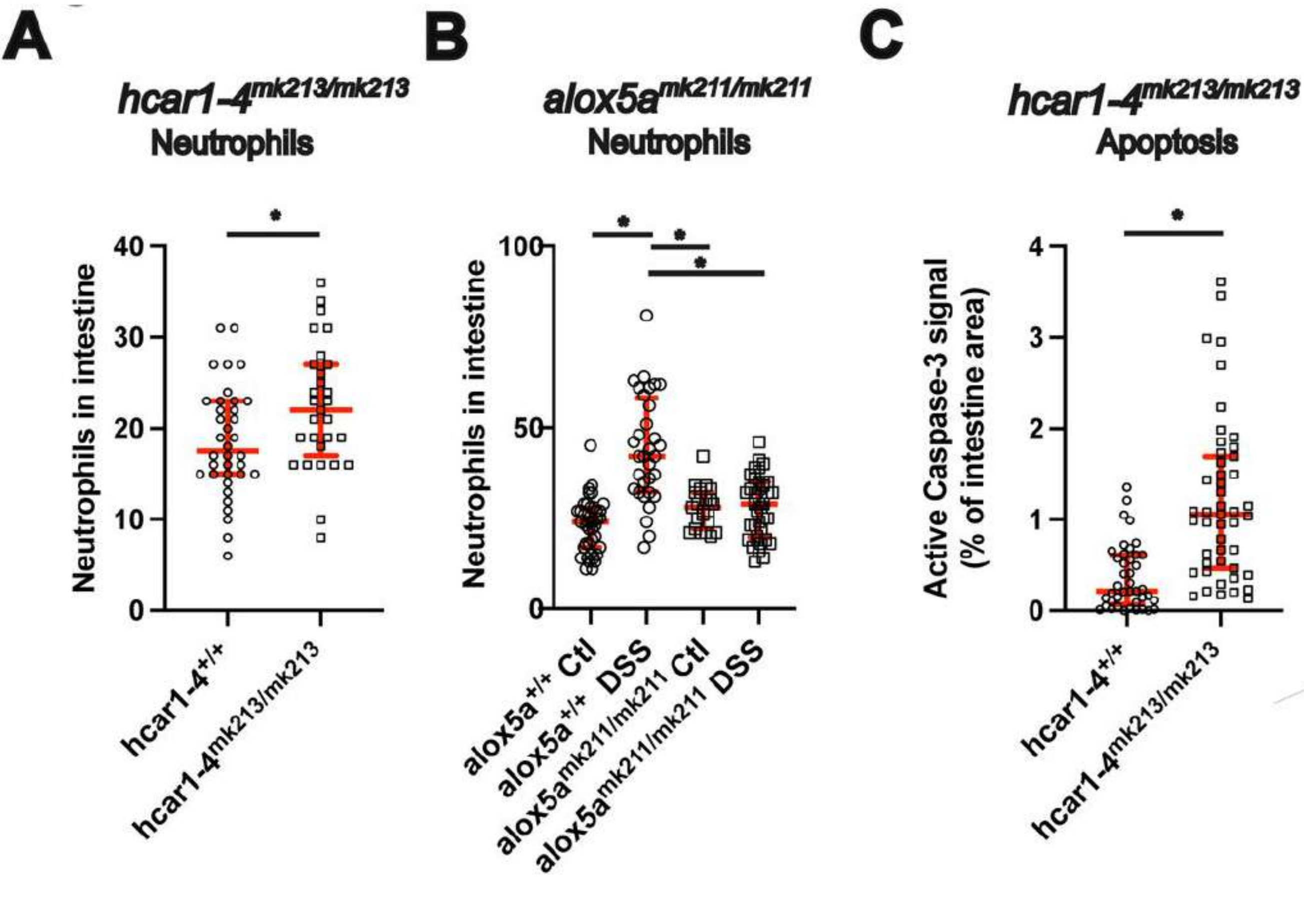
A
hcar1-4mk213/mk213
Neutrophils
Neutrophils in intestine
0
10
20
30
40
*
hcar1-4+/+
hcar1-4mk213/mk213
B
alox5amk211/mk211
Neutrophils
Neutrophils in intestine
0
50
100
*
*
*
alox5a+/+ Ctl
alox5a+/+ DSS
alox5amk211/mk211 Ctl
alox5amk211/mk211 DSS
C
hcar1-4mk213/mk213
Apoptosis
Active Caspase-3 signal
(% of intestine area)
0
1
2
3
4
*
hcar1-4+/+
hcar1-4mk213/mk213

bioRxiv preprint doi: https://doi.org/10.1101/2025.02.05.636712; this version posted February 8, 2025. The copyright holder for this preprint (which was not certified by peer review) is the author/funder, who has granted bioRxiv a license to display the preprint in perpetuity. It is made available under aCC-BY 4.0 International license.

Figure S1. Extended data supporting Figure 1.

(A): Quantification of intestinal neutrophil counts in 6dpf hcar1-4$^{+/+}$ or hcar1-4$^{mk213/mk213}$ larvae, *: p<0.05, Student's t-test (n=29 and 36 larvae). **(B)**: Quantification of intestinal neutrophil counts in 6dpf alox5a$^{+/+}$ or alox5a$^{mk211/mk211}$ larvae after overnight DSS treatment. For the experimental scheme see Figure 1A. *: p<0.05, One-way ANOVA, followed by Tukey's post hoc test (n=23-40 larvae). **(C)**: Quantification of intestinal anti-active caspase-3 immunostaining in 6dpf hcar1-4$^{+/+}$ or hcar1-4$^{mk213/mk213}$ larvae. *: p<0.05, Mann Whitney test (n=41 and 47 larvae)

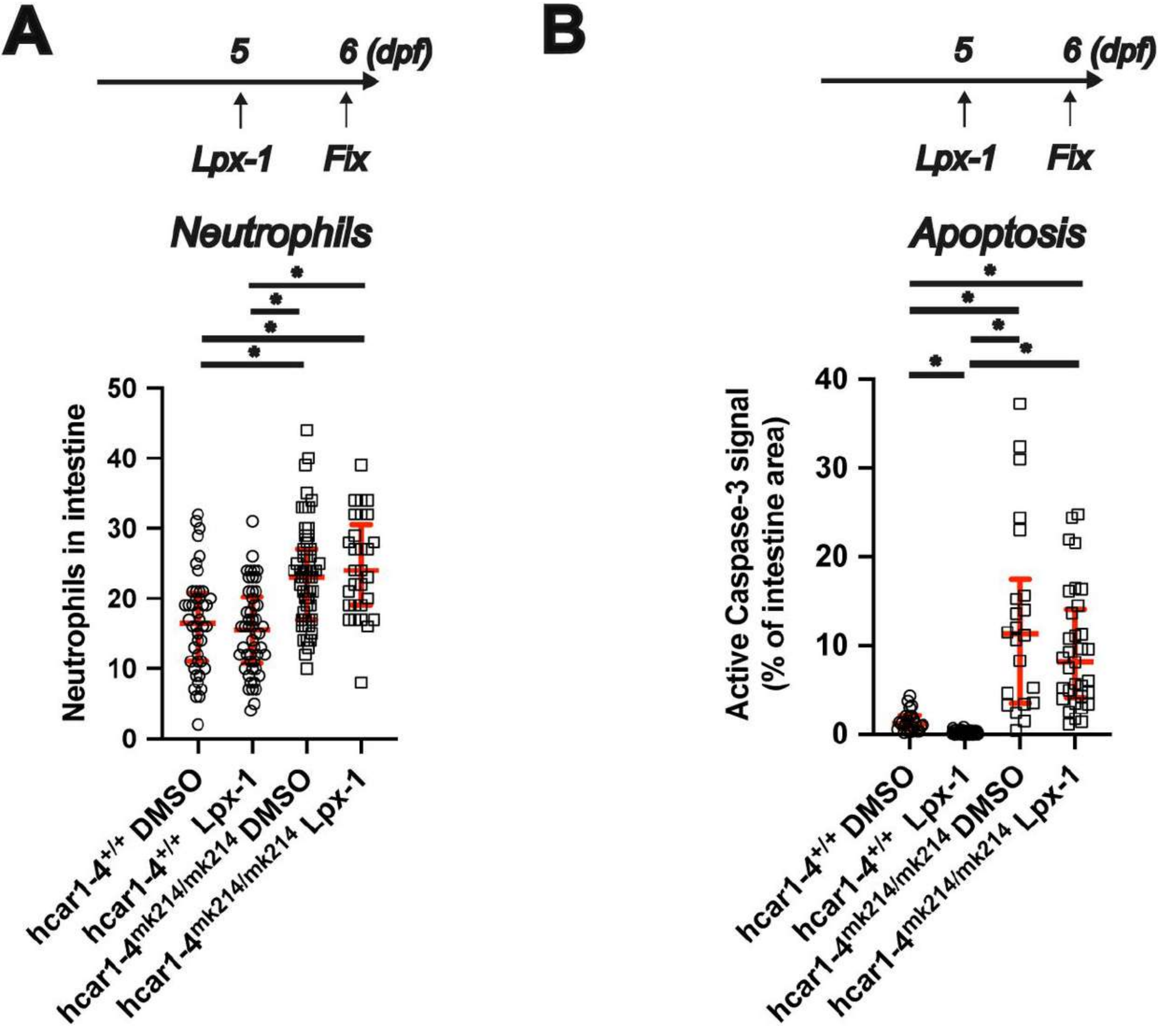
A
5
6 (dpf)
Lpx-1
Fix
Neutrophils
Neutrophils in intestine
50
40
30
20
10
0
hcar1-4+/+ DMSO
hcar1-4+/+ Lpx-1
hcar1-4mk214/mk214 DMSO
hcar1-4mk214/mk214 Lpx-1
B
5
6 (dpf)
Lpx-1
Fix
Apoptosis
Active Caspase-3 signal
(% of intestine area)
40
30
20
10
0
hcar1-4+/+ DMSO
hcar1-4+/+ Lpx-1
hcar1-4mk214/mk214 DMSO
hcar1-4mk214/mk214 Lpx-1

bioRxiv preprint doi: https://doi.org/10.1101/2025.02.05.636712; this version posted February 8, 2025. The copyright holder for this preprint (which was not certified by peer review) is the author/funder, who has granted bioRxiv a license to display the preprint in perpetuity. It is made available under aCC-BY 4.0 International license.

Figure S2. Extended data supporting Figure 2.

(A): Top: Experimental scheme for Liproxstatin-1 treatment. Larvae were treated with 20 μM Liproxstatin-1 (Lpx-1) or DMSO control for 24 hours and fixed at 6dpf for further processing. Bottom: Quantification of neutrophil counts in control and Lpx-1 treated larvae. *: $p<0.05$, One-way ANOVA, followed by Tukey's post hoc test (n=29-55 larvae).**(B)** Top: Experimental scheme for Liproxstatin-1 treatment. Larvae were treated with Liproxstatin-1 (Lpx-1) or DMSO control for 24 hours and fixed at 6dpf for further processing. Bottom: Quantification of intestinal anti-active caspase-3 immunostaining in control and Lpx-1 treated larvae. *: $p<0.05$, Kruskal-Wallis ANOVA, followed by Dunn's post hoc test (n=22-43 larvae).

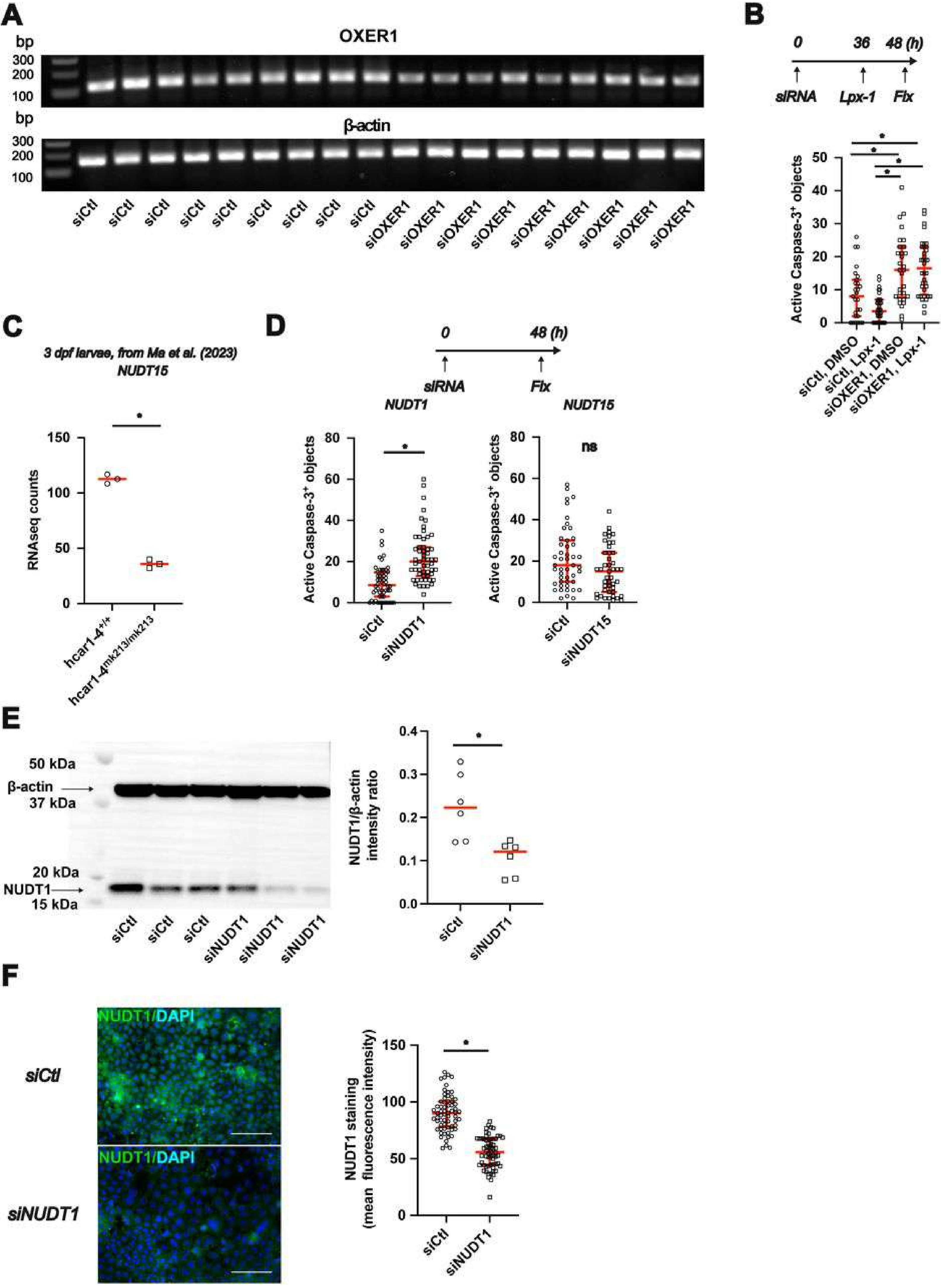
A
bp
OXER1
300
200
100
β-actin
siCtl
siOXER1
B
0
36
48 (h)
siRNA
Lpx-1
Fix
Active Caspase-3+ objects
50
40
30
20
10
0
siCtl, DMSO
siCtl, Lpx-1
siOXER1, DMSO
siOXER1, Lpx-1
C
3 dpf larvae, from Ma et al. (2023)
NUDT15
RNAseq counts
150
100
50
0
hcar1-4+/+
hcar1-4mk213/mk213
D
0
48 (h)
siRNA
Fix
NUDT1
NUDT15
ns
Active Caspase-3+ objects
80
60
40
20
0
siCtl
siNUDT1
siNUDT15
E
50 kDa
β-actin
37 kDa
20 kDa
NUDT1
15 kDa
siCtl
siNUDT1
NUDT1/β-actin intensity ratio
0.4
0.3
0.2
0.1
0.0
F
NUDT1/DAPI
siCtl
siNUDT1
NUDT1 staining (mean fluorescence intensity)
150
100
50
0

bioRxiv preprint doi: https://doi.org/10.1101/2025.02.05.636712; this version posted February 8, 2025. The copyright holder for this preprint (which was not certified by peer review) is the author/funder, who has granted bioRxiv a license to display the preprint in perpetuity. It is made available under aCC-BY 4.0 International license.

Figure S3. Extended data supporting Figure 3.

(A) Caco-2 monolayers were transfected with OXER1 targeting or non-targeting control siRNA. Total RNA was isolated 48 hours after transfection and used for cDNA synthesis. Semiquantitative PCR was performed using primers specific for OXER1 (top) and ß-actin (bottom). **(B)** Top: Experimental scheme. Caco-2 monolayers were transfected with OXER1 targeting or non-targeting control siRNA. Cells were treated with 20 µM Lpx-1 or vehicle control 36 hours after transfection (overnight treatment). Cells were fixed and used for anti-active caspase-3 immunostaining 48 hours after transfection. Bottom: Quantification of apoptosis. *: $p<0.05$, Kruskal-Wallis ANOVA, followed by Dunn's post hoc test (n=27-36 images from N=1 transfection). **(C)**: mRNASeq counts for NUDT15 from 3dpf hcar1-4$^{+/+}$ and hcar1-4$^{mk213/mk213}$ larvae. Replotted from [13], source data deposited in GEO under number GSE201604. **(D)**: Top: Experimental scheme. Caco-2 monolayers were transfected with either non-targeting control siRNA or siRNA targeting NUDT1 or NUDT15. Cells were fixed and used for anti-active caspase-3 immunostaining 48 hours after transfection. Left: Quantification of apoptosis after NUDT1 silencing. *: $p<0.05$, Mann-Whitney test (n=55-56 images from N=2 transfections). Right: Quantification of apoptosis after NUDT15 silencing; $p=0.077$, Mann-Whitney test (n=45-47 images from N=2 transfections). **(E)**: Western blot of Caco-2 cells transfected with either control siRNA or NUDT1 siRNA. Blots were simultaneously probed with anti-ß-actin and anti-NUDT1 antibodies. Arrows mark the expected molecular weight of ß-actin or NUDT1. Right: Quantification of NUDT1 band intensity normalized to the ß-actin band intensity *: $p<0.05$, t-test (n=6-6 samples from N=2 transfections). **(F)**: Caco-2 monolayers were transfected with either non-targeting control siRNA or siRNA targeting NUDT1. Cells were fixed and used for anti-NUDT1 immunostaining 48 hours after transfection. Left: Representative images of anti-NUDT1 immunostaining .Right: quantification of NUDT1immunostaining. *: $p<0.05$, Student's t-test (n=62-65 images from N=2 transfections).

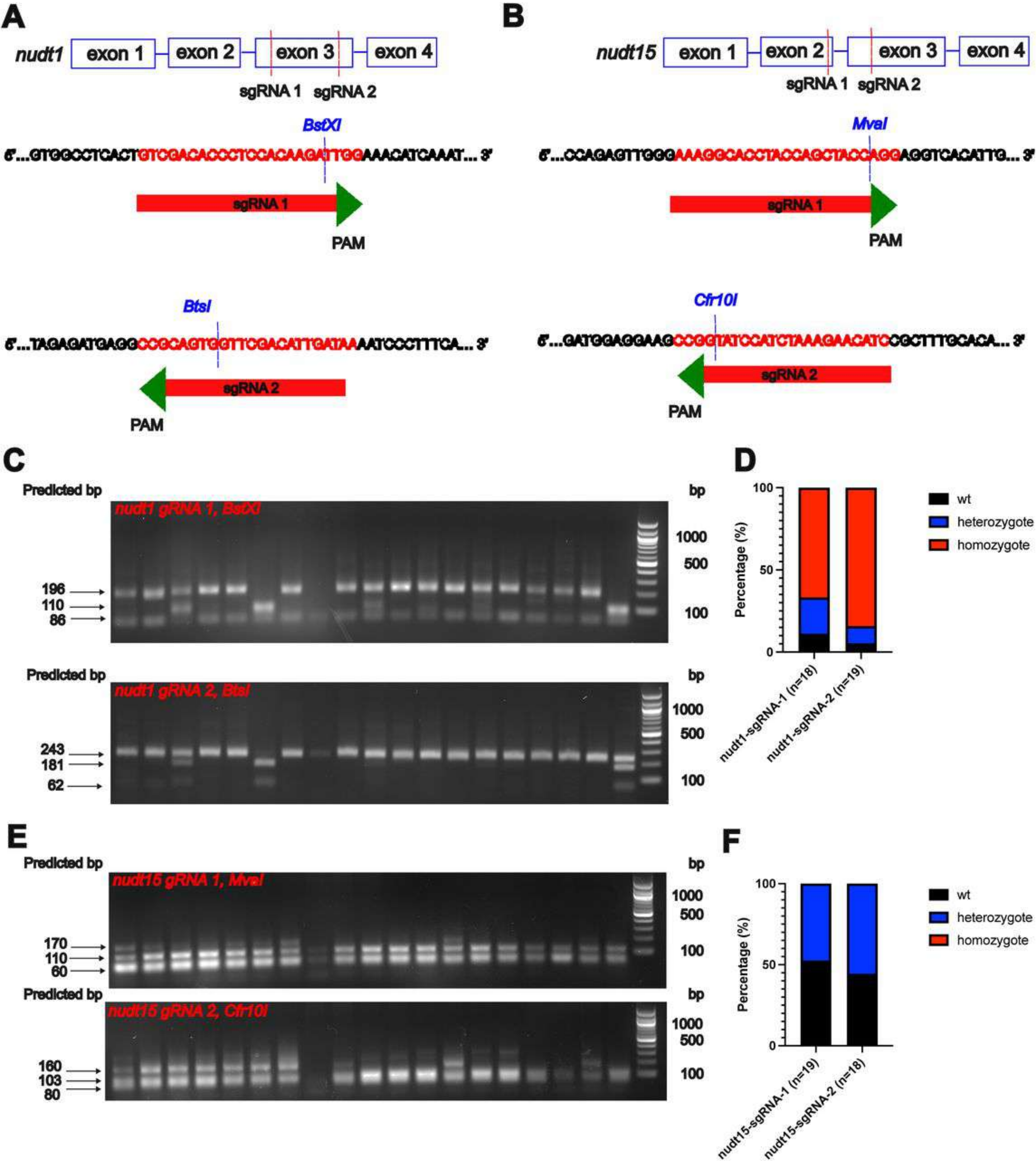
A
nudt1
exon 1
exon 2
exon 3
exon 4
sgRNA 1
sgRNA 2
BstXI
5'...GTGGCCTCACTGTCGACACCCTCCACAAGATTGGAAACATCAAAT... 3'
sgRNA 1
PAM
BtsI
5'...TAGAGATGAGGCCGCAGTGGTTCGACATTGATAAAATCCCTTTCA... 3'
sgRNA 2
PAM
B
nudt15
exon 1
exon 2
exon 3
exon 4
sgRNA 1
sgRNA 2
MvaI
5'...CCAGAGTTGGGAAAGGCACCTACCAGCTACCAGGAGGTCACATTG... 3'
sgRNA 1
PAM
Cfr10I
5'...GATGGAGGAAGCCGGTATCCATCTAAAGAACATCCGCTTTGCACA... 3'
sgRNA 2
PAM
C
Predicted bp
nudt1 gRNA 1, BstXI
196
110
86
bp
1000
500
100
Predicted bp
nudt1 gRNA 2, BtsI
243
181
62
bp
1000
500
100
D
Percentage (%)
100
50
0
nudt1-sgRNA-1 (n=18)
nudt1-sgRNA-2 (n=19)
wt
heterozygote
homozygote
E
Predicted bp
nudt15 gRNA 1, MvaI
170
110
60
bp
1000
500
100
Predicted bp
nudt15 gRNA 2, Cfr10I
160
103
80
bp
1000
500
100
F
Percentage (%)
100
50
0
nudt15-sgRNA-1 (n=19)
nudt15-sgRNA-2 (n=18)
wt
heterozygote
homozygote

bioRxiv preprint doi: https://doi.org/10.1101/2025.02.05.636712; this version posted February 8, 2025. The copyright holder for this preprint (which was not certified by peer review) is the author/funder, who has granted bioRxiv a license to display the preprint in perpetuity. It is made available under aCC-BY 4.0 International license.

Figure S4. Extended data supporting Figure 4.

(A, B) Schematic representation of zebrafish *nudt1* and *nudt15*. 2 guide RNAs (red text) were designed to disrupt exon2 (*nudt1)* and exons 2 and 3 (*nudt15*) and to disrupt restriction enzyme recognition sites (cleavage site marked with a blue dash) in these genes. **(C, E)** *nudt1* and *nudt15* F0 CRISPR larvae (18 or 19 per mutant) were genotyped by PCR amplification, restriction analysis, and agarose gel electrophoresis. For *nudt1* guide RNA-1, the digest of the wild type samples yields two lower bands of 110 and 86bp, digested heterozygotes have an additional higher band of 196 bp and homozygotes are uncleaved and result in only the 196bp band. For *nudt1* guide RNA-2, the digest of wild type samples results in two bands of 181 and 62 bp, heterozygotes have an additional higher band of 243 bp, while PCR products from homozygotes are uncleaved and only have the 243 bp band. For *nudt15* guide RNA-1, digested PCR products from wild type samples yield two bands of 100 and 60 bp, heterozygotes have an additional 170bp band, while in the case of the homozygotes, only the 170bp band is visible. For *nudt15* guide RNA-2, digested PCR products from wild type samples yield two bands of 103 and 80 bp (with an additional 57 bp fragment that can't be visualized properly), heterozygotes have an additional 160 bp band, while in the case of the homozygotes, only the 160bp and 80bp band is visible. **(D, F)** The fraction of F0 CRISPR homozygotes, heterozygotes, or wt animals among the 18 or 19 tested larvae in C and D.

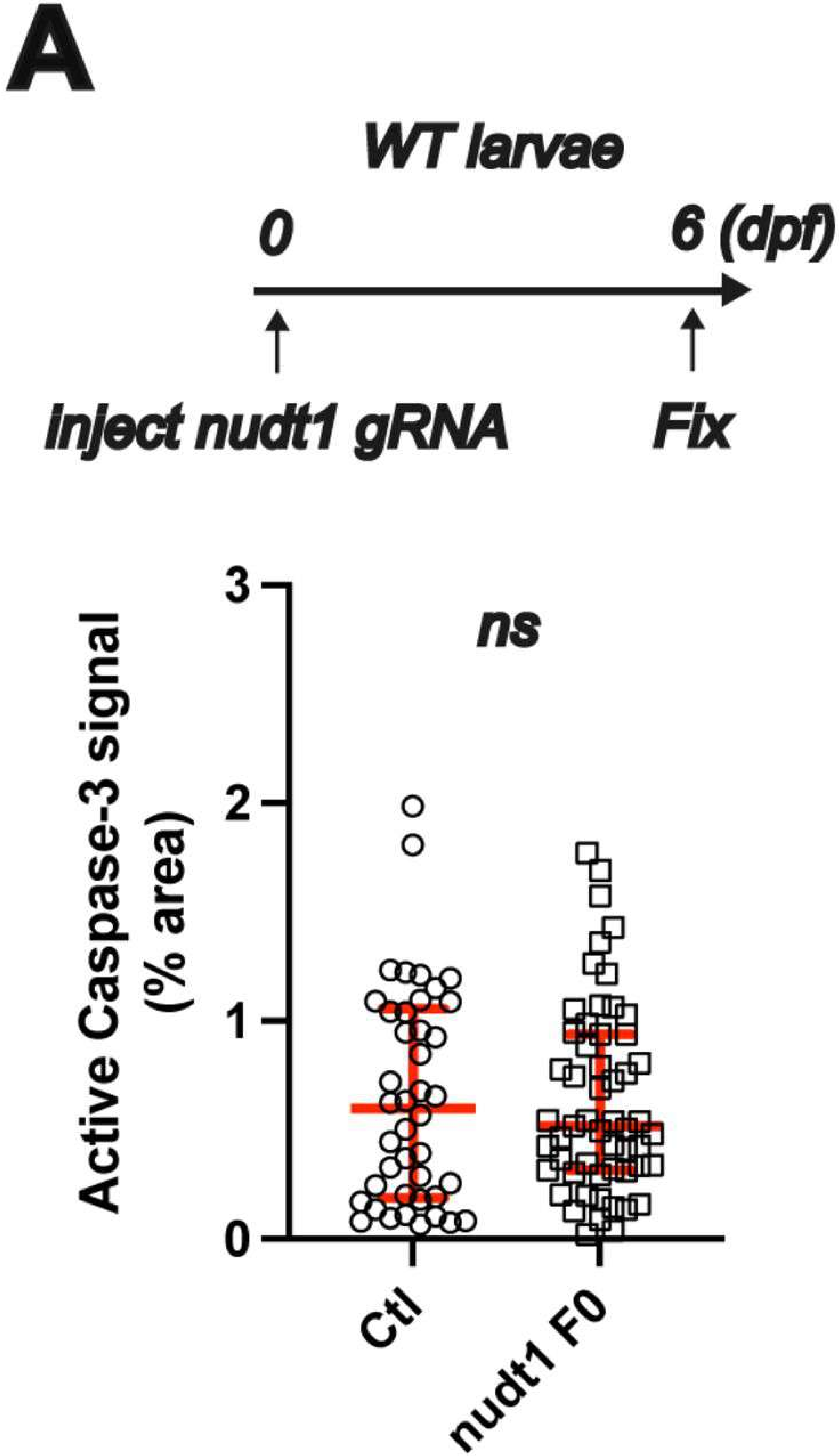
A
WT larvae
0
6 (dpf)
inject nudt1 gRNA
Fix
Active Caspase-3 signal
(% area)
3
2
1
0
ns
Ctl
nudt1 F0

bioRxiv preprint doi: https://doi.org/10.1101/2025.02.05.636712; this version posted February 8, 2025. The copyright holder for this preprint (which was not certified by peer review) is the author/funder, who has granted bioRxiv a license to display the preprint in perpetuity. It is made available under aCC-BY 4.0 International license.

Figure S5. Extended data supporting Figure 4.

(A) Top: Experimental scheme. WT zebrafish embryos were injected with Cas9 and guide RNAs *for nudt1* at the one cell stage. Larvae were fixed at 6dpf and used for anti-active caspase-3 immunostaining. Bottom: Quantification of apoptosis in control and F0 injected larvae. *Nudt1* F0 CRISPR had no effect on intestinal epithelial apoptosis; p=0.85, Mann-Whitney test (n= 42 and 55 larvae).
